# Multi-targeted properties of the probiotic *saccharomyces cerevisiae* CNCM I-3856 against enterotoxigenic *escherichia coli* (ETEC) H10407 pathogenesis across human gut models

**DOI:** 10.1080/19490976.2021.1953246

**Published:** 2021-08-25

**Authors:** Charlène Roussel, Kim De Paepe, Wessam Galia, Jana de Bodt, Sandrine Chalancon, Sylvain Denis, Françoise Leriche, Pascal Vandekerkove, Nathalie Ballet, Stéphanie Blanquet-Diot, Tom Van de Wiele

**Affiliations:** a Université Clermont Auvergne, UMR UCA-INRA 454 MEDIS, Microbiology Digestive Environment and Health, Clermont-Ferrand, France; b CMET, Center for Microbial Ecology and Technology, Department of Biotechnology, Faculty of Bioscience Engineering, Ghent University, Ghent, Belgium; c UMR 5557 Microbial Ecology, Research Group On Bacterial Opportunistic Pathogens And Environment, CNRS, VetAgro Sup, Lyon, France; d Unité De Recherche Fromagère, VetAgro Sup, Lempdes, France; e Lesaffre International, Lesaffre Group, Marcq-en-Baroeul, France

**Keywords:** Probiotic, *Saccharomyces cerevisiae*, foodborne pathogen, ETEC; virulence, enterotoxin, antagonism effect, gut microbiota

## Abstract

Enterotoxigenic *Escherichia coli* (ETEC) is one of the most common causes of acute traveler’s diarrhea. Adhesins and enterotoxins constitute the major ETEC virulence traits. With the dramatic increase in antibiotic resistance, probiotics are considered a wholesome alternative to prevent or treat ETEC infections. Here, we examined the antimicrobial properties of the probiotic *Saccharomyces cerevisiae* CNCM I-3856 against ETEC H10407 pathogenesis upon co-administration in the TNO gastrointestinal Model (TIM-1), simulating the physicochemical and enzymatic conditions of the human upper digestive tract and preventive treatment in the Mucosal Simulator of the Human Intestinal Microbial Ecosystem (M-SHIME), integrating microbial populations of the ileum and ascending colon. Interindividual variability was assessed by separate M-SHIME experiments with microbiota from six human individuals. The probiotic did not affect ETEC survival along the digestive tract. However, ETEC pathogenicity was significantly reduced: enterotoxin encoding virulence genes were repressed, especially in the TIM-1 system, and a lower enterotoxin production was noted. M-SHIME experiments revealed that 18-days probiotic treatment stimulate the growth of *Bifidobacterium* and *Lactobacillus* in different gut regions (mucosal and luminal, ileum and ascending colon) while a stronger metabolic activity was noted in terms of short-chain fatty acids (acetate, propionate, and butyrate) and ethanol production. Moreover, the probiotic pre-treated microbiota displayed a higher robustness in composition following ETEC challenge compared to the control condition. We thus demonstrated the multi-inhibitory properties of the probiotic *S. cerevisiae* CNCM I-3856 against ETEC in the overall simulated human digestive tract, regardless of the inherent variability across individuals in the M-SHIME.

## Introduction

The number of diarrheal diseases related to enterotoxigenic *Escherichia coli* (ETEC) amounts to 44 million cases annually and represents one of the leading causes of morbidity in countries or regions with limited access to resources, such as safe drinking water^1^. ETEC is also one of the most frequent bacterial causes of diarrhea for people traveling in damp tropical regions, including military personnel deployed in these areas.^2,3^ Sporadically, such infections extend to industrialized nations of temperate regions.^4^ ETEC virulence is initiated by a large set of colonization factors and adhesins (e.g., CFA/I, FimH) mediating attachment to the intestinal epithelial cells in distal part of the small intestine. The subsequent release of heat labile (LT) and/or heat stable (ST) enterotoxins leads to the onset of profuse watery diarrhea and consecutive dehydration.^5^

ETEC infections are treated according to general clinical recommendations applicable to diarrheal episodes with standard treatment of care including antibiotic therapy in the case of acute diarrhea.^6–8^ Prophylactic strategies or therapies specifically targeting ETEC pathogenesis are currently not available on the market. The lack of such targeted approach, the absence of successful preventive measures against ETEC and moreover the dramatic increase of antibiotic-resistance and its concomitant health repercussions further challenge the health-care system,^9,10^ highlighting the importance to expedite the development of prophylactic natural approaches. In this context, probiotics are considered a wholesome alternative.

The last two decades of probiotic research have established scientific support for the use of bacterial and yeast probiotic strains by showing interference with ETEC survival, adhesion to mucins and/or expression of virulence genes.^5^ However, most of these studies were carried out in piglets with ETEC F4^+^ or F5^+^ strains that are strongly associated with neonatal and post-weaning enteric colibacillosis in animals, but unlikely to be pathogenic in humans.^11,12^ Health effects from bacterial pathogens are strain dependent. Therefore, assessing probiotic strategies against human ETEC pathotypes requires studies on human strains with an experimental setup that is representative for the human gut. For obvious reasons, reports on human trials are limited. Only two human clinical trials have investigated the prophylactic effect of probiotics when orally challenged with live attenuated human ETEC strains.^13,14^ Unfortunately, the use of a single strain of *Lactobacillus acidophilus* or a blend of probiotic bacteria and yeast in these studies failed to provide convincing evidence to use probiotics in the prevention of ETEC-associated symptoms. Other studies with probiotics administered prophylactically such as *Saccharomyces cerevisiae var. boulardii* have shown a significant reduction in the risk of contracting traveler’s diarrhea, yet the etiology behind this protective effect remains unknown.^15,16^ An expert panel of the International Society of Travel Medicine concluded that evidence is insufficiently available to recommend the use of commercial pre- or probiotics to prevent or treat traveler’s diarrhea.^16^ We previously set out to investigate the anti-infectious properties of the probiotic *Saccharomyces cerevisiae* CNCM I-3856 against the human ETEC reference strain H10407.^17^ This probiotic was found to display beneficial effects against enteric *E. coli* pathogens^18–20^ including ETEC H10407 in *in vitro* batch cultures, intestinal epithelial cell cultures and in an *in vivo* mouse model.^17^

The purpose of the present study was to investigate the modulatory effects of the probiotic *S. cerevisiae* CNCM I-3856 on the dynamics of ETEC H10407 survival, physiological state and its virulence features, along successive human gastrointestinal niches from stomach to ascending colon simulated by using complementary *in vitro* digestive models, the TNO gastrointestinal Models (TIM-1) and Mucosal Simulator of the Human Intestinal Microbial Ecosystem (M-SHIME). The capacity of the probiotic to shape the functionality and composition of the gut microbial ecosystems was explored by means of next-generation *16S* rRNA gene amplicon sequencing.

## Results

### Probiotic treatment has no direct inhibitory effect on ETEC survival in the in vitro gut

**Figure 1.**
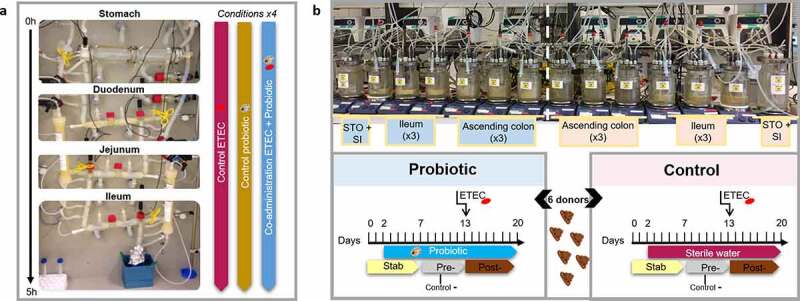
TIM-1 and M-SHIME set-up. (a) Picture of the TIM-1. Gastro-intestinal digestions (without microbiota) were performed in 3 separated conditions, in quadruplicate: (i) control experiment consisted of feeding the stomach compartment with mineral water (200 mL) experimentally contaminated with ETEC (7.5 log_10_ CFU mL^−1^); (ii) probiotic control condition consisted in the administration of the yeast *S. cerevisiae* alone (7.5 log_10_ CFU mL^−1^); and (iii) probiotic treatment condition consisted in the co-administration of ETEC (7.5 log_10_ CFU mL^−1^) and the probiotic yeast (7.5 log_10_ CFU mL^−1^). (b) Picture of the M-SHIME system mimicking the digestive and fermentative conditions. The stomach (STO) /combined duodenum-jejunum (SI) vessel was connected to three ileum bioreactors coupled to three respective ascending colon vessels. The run was performed twice (in total six distinct microbiota from healthy individuals). Each microbiota has been cultivated in parallel under 2 conditions, probiotic *vs* control. Probiotic treatment consisted of yeast *S. cerevisiae* CNCM I-3856 resuspended in 30 mL sterile water (7.5 log_10_ CFU mL^−1^) and added in the SHIME stomach, twice a day (9 a.m and 5 p.m) during 18-days from day 2 to 20. Under control condition, a sham treatment with 30 mL sterile water was performed during day 2 to 20. In addition, ETEC challenge was tested under both conditions, and the pre- (negative ETEC control) *vs* post-ETEC (positive) infection were discriminated as following: (i) the days 7 to 12 were kept as pre-infection period; while (ii) at day 13, both control and probiotic treated systems were challenged with ETEC by inoculation of 7.5 log_10_ CFU mL^−1^ in SHIME ileum vessels, defining days 13 to 20 as post-infectious period

**Figure 2.**
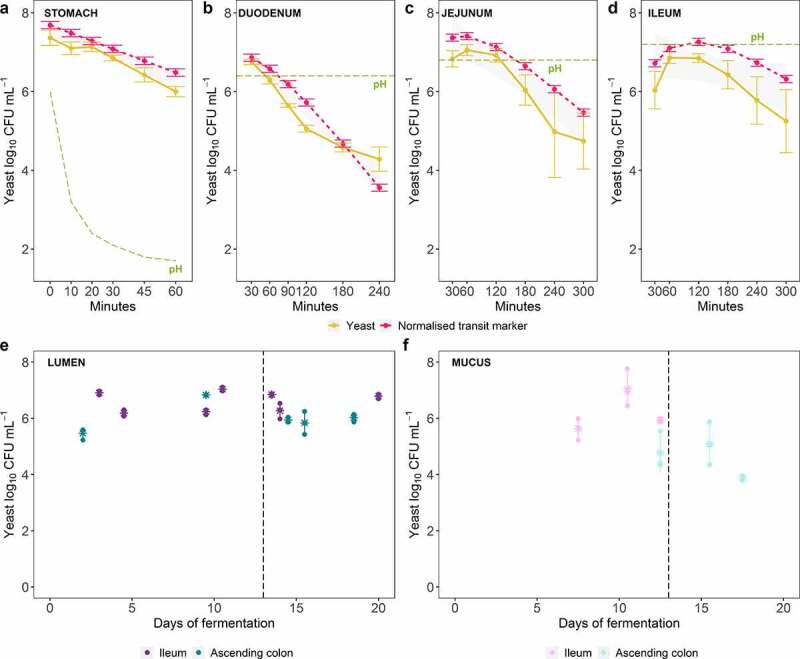
Dynamics of the probiotic yeast *S. cerevisiae* survival along the *in vitro* human gastrointestinal tract. (a, b, c, d) Average of the probiotic yeast log_10_ CFU mL^−1^ ± SD (n = 4) (in orange), determined by plate counts in each gastrointestinal region reproduced by TIM-1 system compared with an inert non-absorbable transit marker indicating 100% survival (in pink). Probiotic curves below that of the transit marker reflect cell mortality, while curves above the transit marker are indicative of a growth. The level of the pH in each region is indicated by a green dashed line. No statistically significant difference over time between the probiotic and the transit marker was found, as determined by pairwise wilcoxon rank sum tests with holm correction. Confidence level intervals (95%) are displayed in gray. (e, f) The number of probiotic yeast log_10_ CFU mL^−1^ ± SD, determined by plate counts, over 18-days fermentation in each microbial niche reproduced by M-SHIME system. The experiment was repeated with the fecal microbial communities from six different healthy donors. Average of these biological replicates is indicated with a ‘*’ and the SD is shown. No statistically significant difference over time between the probiotic and the transit marker was found, as determined by pairwise wilcoxon rank sum tests with holm correction

The overall study design from the two complementary models TIM-1 and M-SHIME is presented in Figure 1. The fate of the probiotic *S. cerevisiae* was followed up in the upper (Figure 2 (a-d)) and lower simulated gastrointestinal tract (Figure 2(e-f)) by plate counts. Probiotic survival in TIM-1 was not impacted by gastrointestinal stressors, such as low gastric pH or bile salts (Figure 2(a-d)). In M-SHIME, administration of the probiotic at a dose of 7.5 log_10_ mL^−1^ twice a day was sufficient to maintain a concentration between 5 and 6 log_10_ mL^−1^ over time, in mucosal and luminal regions of ileum and ascending colon (Figure 2(e-f)).

**Figure 3.**
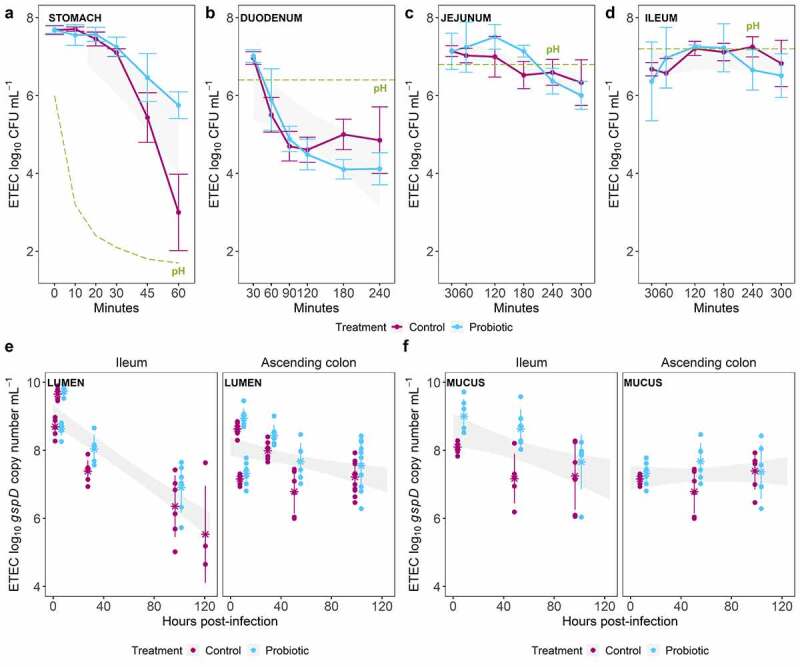
Effect of the probiotic treatment on ETEC survival along the *in vitro* human gastrointestinal tract over time. (a, b, c, d) Average of the ETEC log_10_ CFU mL^−1^ ± SD (n = 4) (in purple), determined by plate counts in each gastrointestinal region reproduced by TIM-1 system compared with ETEC co-administered with the probiotic *S. cerevisiae* (in blue). The level of the pH in each region (stomach, duodenum, jejunum and ileum) is indicated by a green dashed line. (e, f) The ETEC log_10_ *gspD* copy number mL^−1^ (in purple), determined by qPCR, in each microbial niche reproduced by the M-SHIME system, compared with ETEC administered to a continuous probiotic-treated microbial community (in blue). The experiment was repeated with the fecal microbial communities from six different healthy donors. The average of these biological replicates is indicated with a ‘*’ and the SD is shown. No statistically significant difference over time between the control and probiotic conditions was found, as determined by pairwise wilcoxon rank sum tests with holm correction. Confidence level intervals (95%) are displayed in gray, in each figure panel

Plate count-based analysis of gastric or intestinal effluents from TIM-1 revealed no significant difference in ETEC survival when *S. cerevisiae* CNCM I-3856 was co-administered at 7.4 log_10_ CFU mL^−1^ or not (Figure 3(a-d)). ETEC was neither affected by *S*. cerevisiae in presence of a simulated human gut microbiota. Targeting the ETEC specific *gspD* gene to cope with the complexity of a background microbiota, qPCR analysis of luminal and mucosal samples from terminal ileum and proximal colon regions from M-SHIME did not show ETEC to be negatively affected by continuous probiotic treatment (Figure 3(e-f)). This was biologically reproducible across experiments with microbiota from all six donors. Altogether, these results suggest that the probiotic yeast does not elicit a direct inhibitory effect toward ETEC survival.

### Probiotic treatment affected ETEC membrane integrity in the upper digestive tract

**Figure 4.**
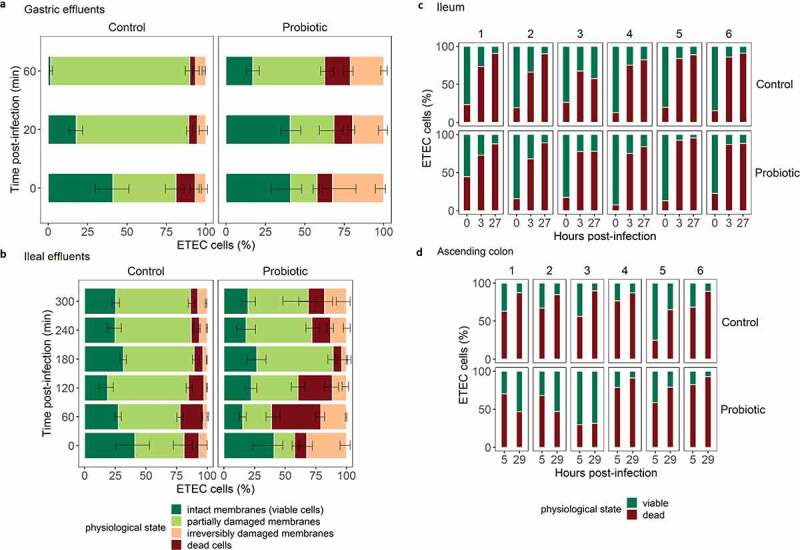
Effect of the probiotic treatment on ETEC physiological states in the *in vitro* human gastrointestinal tract over time. (a, b) ETEC membrane integrity was measured by flow cytometry of live/dead stained samples in TIM-1. Four subpopulations were discriminated after SYTO9 and PI staining: intact membranes (green), partially damaged membranes (pale green), irreversibly damaged membranes (pale pink) and dead cells (red). The bar plots represent the average percentages from two independent replicates over time under control and probiotic conditions. (c, d) ETEC membrane integrity was measured by PMA-qPCR on the *gspD* gene 5 and 29 h post ETEC infection (day 13) in the M-SHIME. The number of dead cells was obtained after deducting the number of viable cells from the total ETEC cell count measured by qPCR. The bar plots in percentages are represented for each donor (1 to 6) under both control and probiotic treatment conditions

Viability staining and subsequent flow cytometry analysis of TIM-1 samples revealed four different ETEC subpopulations. By gating on the cytogram, ETEC cells were distinguished from the probiotic *S*. cerevisiae cells when co-existing in the same medium (Supplementary Data Figure S1). As viability staining primarily depends on bacterial cell wall permeability, these subpopulations reflect the degree of damage of ETEC cells (Figure 4 (a-b)). The probiotic treatment tended to increase the number of damaged and dead ETEC cells over time compared to the control condition, both for gastric and ileal effluents (Figure 4 (a-b)). Gastric effluents displayed a clear shift from viable/damaged cells under control conditions to damaged/dead cells under probiotic condition (Figure 4a). Consequently, at the end of the gastrointestinal digestion (300 min in the ileal effluents), about 25% of the total ETEC cells entering the colon had an intact membrane under control conditions, compared to 19% under probiotic supplementation (Figure 4b). In luminal phase of the M-SHIME, due to complex microbial background, ETEC specific PMA-qPCR was applied to analyze ETEC viability (Figure 4c-d). Averaging the results from all six M-SHIME runs, no difference in live/death ratios for ETEC from ileum and ascending colon samples were found between probiotic treatment and control (Figure 4c-d). Clear interindividual differences were, however, noted. M-SHIME ileum from donor 3 and 5 displayed a clear drop in ETEC viability under the probiotic condition. For instance, a fall of 22% ETEC viability was found in donor 3, 27 h post-infection (Figure 4c). Yet, this decrease in viability was not consistent toward the ascending colon. For instance, the probiotic treatment favors the number of viable ETEC in donor 1 and 3, 29 h post-infection (Figure 4d).

ETEC membrane potential was followed up by flow cytometry with DiOC_2_(3) staining in TIM-1 (Table 1). In response to probiotic treatment, ETEC membranes were less polarized in gastric effluents. A sharp depolarization was found after 60 min gastrointestinal digestion in ileal effluents under probiotic treatment, in comparison with control condition (Table 1). Intracellular pH of ETEC cells in all gastrointestinal regions simulated by TIM-1 was not affected by probiotic amendment (Supplementary Data Table S1).

**Table 1.** Effect of the probiotic treatment on ETEC membrane potential in TIM-1 system. According to the fluorescence intensity (FI) ratio, membranes are considered to be depolarized (FI ratio approaching 1) and polarized (FI ratio exceeding 1.2). The table shows the mean of two independent replicates ± SD under control and probiotic treatment conditions

Digestive compartment	Time point (min)	FI ratio sample/control	FI ratio sample/treatment
**Inoculum**	0	1.39 ± 0.58	1.43 ± 0.15
**Gastric effluents**	20	1.78 ± 0.47	1.05 ± 0.79
	60	1.33 ± 0.56	0.99 ± 0.07
**Ileal effluents**	60	2.52 ± 0.65	0.49 ± 0.05
	120	2.45 ± 0.14	1.79 ± 0.15
	180	1.56 ± 0.09	1.44 ± 0.08
	240	1.27 ± 0.07	1.63 ± 0.24
	300	1.40 ± 0.02	1.21 ± 0.22

### Probiotic treatment affects ETEC-virulence

**Figure 5.**
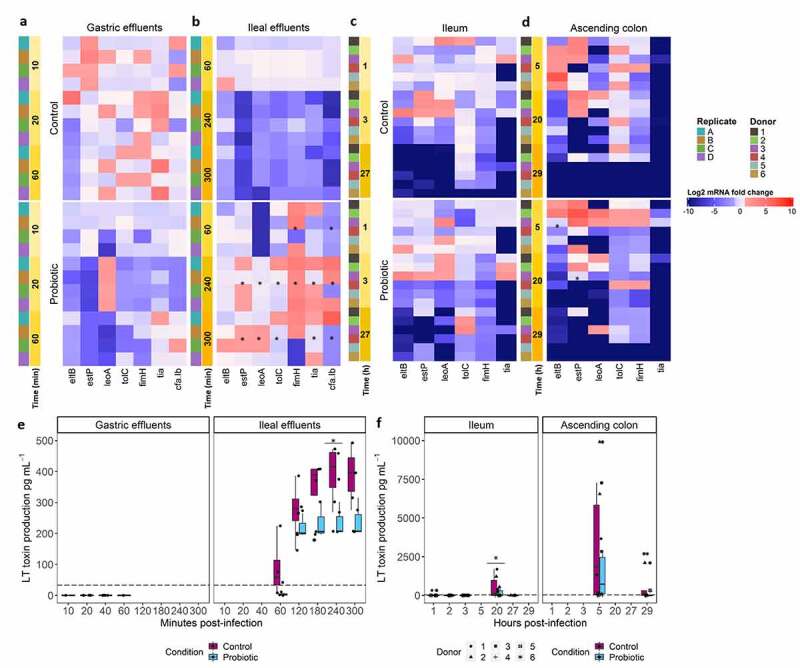
Effect of the probiotic treatment on ETEC virulence gene expression and LT toxin production in the *in vitro* gastrointestinal tract over time. (a, b, c, d) ETEC virulence genes are expressed and colored according to the log_2_ fold-change as determined by RT-qPCR. Statistically significant differential expression between control and probiotic conditions are indicated when a mean log_2_ fold change expression is ≥ 1 (induction denoted in shade of pink) or ≤ −1 (repression denoted in shade of blue), and a *p* ≤ 0.05 (*), as determined by the Friedman post-hoc nemenyi test. Results of all (a, b) four replicates are shown in the gastric and ileal effluents of TIM-1, and (c, d) six donors in luminal ileum and ascending colon of M-SHIME. (e, f) LT toxin production was measured by ELISA and expressed in pg mL^−1^ ± SD (e) as a mean of four independent replicates in TIM-1 and, (f) for each of the six donors in luminal niche of M-SHIME ileum and ascending colon. The black dashed line represents the detection limit. Statistically significant differences in LT toxin production between the control and probiotic conditions are denoted at *p* < .05 (*), as determined by the Friedman post-hoc fisher test

The expression of ETEC H10407 virulence genes was monitored in the simulated upper digestive tract with TIM-1 (Figure 5(a-b)) and in the lower digestive tract lumen with M-SHIME (Figure 5(c-d)). By averaging replicates/donors, we compared over time the statistical difference of log_2_ fold changes (log_2_FC) in control *versus* probiotic treatment for genes encoding for attachment and colonization factors (cfa/Ib, tia, fimH), enterotoxin production (eltB, estP) and enterotoxin release (leoA, tolC) (Figure 5).

Simulation of the upper digestive tract in TIM-1 resulted in most of the virulence genes to be significantly repressed during gastric conditions with probiotic (Figure 5a), while ileal digestion with probiotic displayed an overexpression of virulence genes (Figure 5b). The genes encoding for ETEC adhesins, *fimH*, *tia*, *cfa/Ib* genes were all significantly repressed in gastric effluents under probiotic condition, and significantly over-expressed in ileal effluents (p < .05). Particularly, *fimH* gene encoding type 1 pili, exhibited a 3.6-fold induction under probiotic treatment until 240 min, compared to a − 1.7-fold repression under control condition (p < .05) (Figure 5b).

The *estP* gene was repressed over time (log_2_FC = −4) in stomach under probiotic treatment while over-expressed the 10 first minutes (log_2_FC = 5.5, p < .05) under control condition. An opposite profile was found at 240 and 300 min in ileal effluents with an over-expression of *estP* under probiotic treatment (log_2_FC = 1, p < .05) (Figure 5 (a-b)). Under probiotic treatment no significant change was observed for *eltB* gene encoding for the LT toxin, while the *leoA* gene expression, encoding for delivery of that toxin was variable over time. The gene was significantly over-expressed under probiotic treatment at 20 min in gastric effluents and 300 min in ileal effluents, (log_2_FC = 1.5, p < .05) (Figure 5(a-b)).

In the M-SHIME (Figure 5(c-d)), a strong donor-variability was found, resulting in few statistically significant differences between probiotic and control conditions over time. In ileum (Figure 5c), the *eltB*, *leoA*, *tia*, and *fimH* gene expression patterns were unchanged between control and probiotic conditions. Three hours post-infection in ileum, although not statistically different, the *estP* gene was over-expressed is many donors under control condition (donors 1, 2 and 3, mean log_2_FC = 2.3) but only in one donor, the donor 3 (log_2_FC = 1.3), under probiotic treatment (Figure 5c). This was also observed in ascending colon (Figure 5d), 20 h post-infection where the *estP* gene was significantly more expressed (log_2_FC = 3, p < .05) for 3 out of 6 donors (donors 1, 2, 3) under control condition. The *eltB* gene also displayed a significantly reduced expression in ascending colon 5 h post-infection under probiotic treatment for two donors (donors 1 and 2) with a 1.9-fold induction, while five donors exhibited a mean of 3.5-fold over-expression under control condition (*p < .05*) (Figure 5d). Interestingly, the two donors 2 and 3 displayed simultaneous over-expression of the *estP, leoA, tolC* and *fimH* genes 5 h post-infection in ascending colon with probiotic. Finally, *cfa/Ib* mRNA was not detectable (not amplified) in both ileum and ascending colon contrary to the TIM-1 system (Figure 5).

### Probiotic treatment affects LT toxin production

No production of LT toxin was found upon gastric digestion, neither under control nor probiotic conditions (Figure 5e). The probiotic treatment generally tends to reduce LT toxin production in cumulated ileal effluents, although variability between replicate experiments was noted. At 240 min gastrointestinal digestion, a significant decrease (*p =* .044) was found, with a mean production of 393 ± 114 pg mL^−1^ and 238 ± 62 pg mL^−1^ under control and probiotic conditions, respectively (Figure 5e). In M-SHIME ileum under probiotic condition, an approximate 4-fold decrease of LT was measured 20 h post-infection in ileum for all donors that originally exhibited a high production under control condition (donors 1, 2, and 3, *p =* .048) (Figure 5f). In ascending colon, LT toxin levels were highly variable between donors. Upon probiotic treatment 5 h post-infection, a 3-fold decrease was found for donors 1, 3, and 4, with a decrease from 3660 to 1315 pg mL^−1^ for donor 3 (Figure 5f). As an exception, donor 2 was the only one responding in an adverse way to probiotic treatment with an increase in LT toxin production up to 9910 pg mL^−1^ 5 h post-infection in ascending colon compared to control condition.

### Probiotic treatment induces significant changes in the microbial community structure in a niche-dependent way

**Figure 6.**
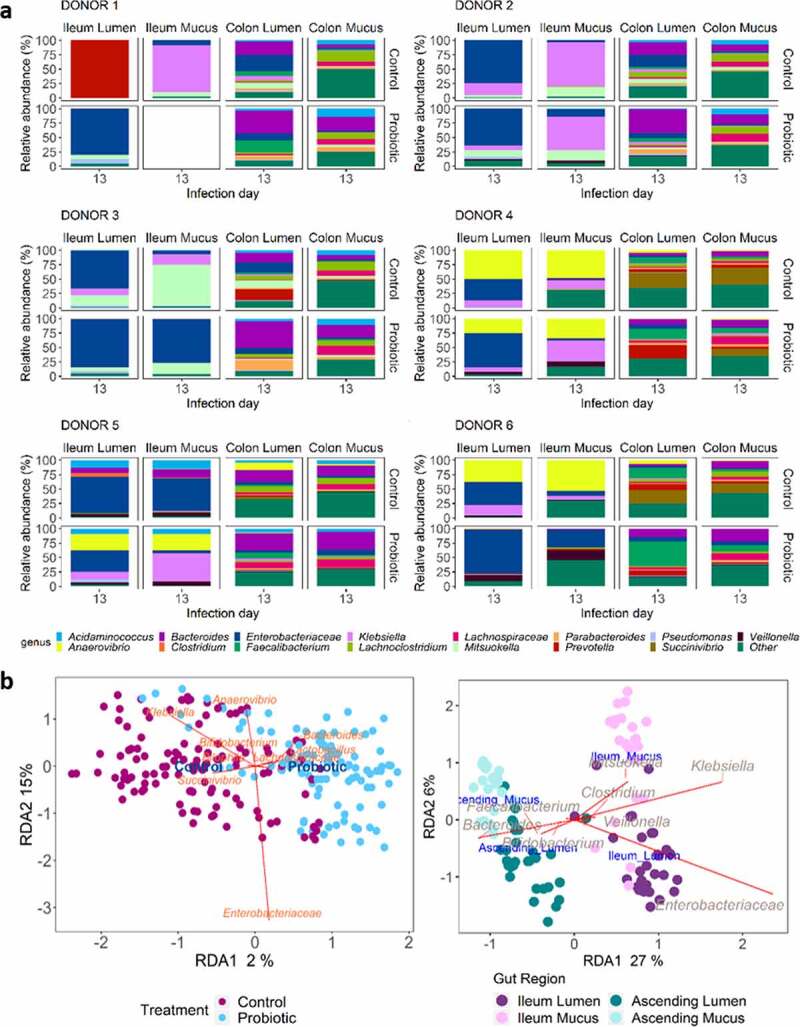
Microbial community composition in the M-SHIME according to the donor, gut region, and the probiotic treatment. (a) Genus level relative abundance of the microbial community composition at day 13 (day of ETEC infection) for all donors according to the gut region and treatment condition, as determined by 16S rRNA gene amplicon sequencing. (b) Triplots showing the significant effects of the treatment condition (R^2^adjusted = 5.2%, *p = *.001***) and gut regions (R^2^ adjusted = 19.5%, *p = *.001***) as main explanatory variables to the microbial community structure at genus level, as determined by distance-based redundancy analysis (db-RDA)

**Figure 7.**
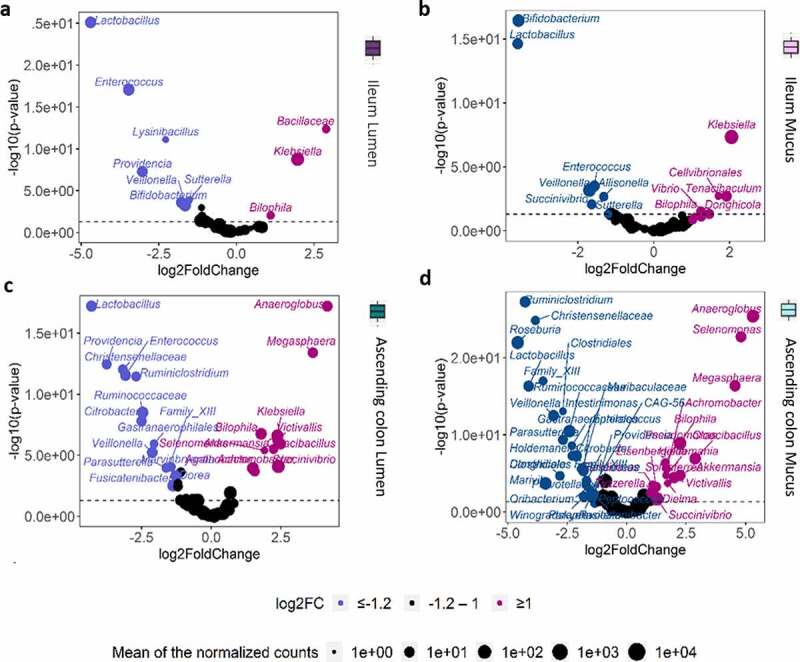
Volcano plots indicating the genera significantly enriched by the probiotic treatment compared to the control in each gut niche over the course of the 20 days lasting M-SHIME run. Analysis was performed from day 7 (stabilized microbiota) to 20. A positive log_2_ fold-change indicates a stimulation of the genus in the control condition (in purple) compared to the probiotic condition (in blue). The log transformed adjusted *p*-value is displayed on the y-axis and the α = .05 significance level is indicated by a dashed line. Only the genera with an absolute log_2_ fold-change value exceeding 1.2 are represented, as determined by deseq2 analysis

The evolution of the microbial community composition subjected to ETEC infection (day 13) was assessed in M-SHIME, under control and probiotic treatment by considering the overall time-period from day 7 to 20. The microbial community at phylum, genus, and OTU level was affected by probiotic amendment in a donor and niche-specific way (Figure 6a, Supplementary Data Figure S2-7). The significant probiotic effect on the overall microbial community structure was confirmed by a distance-based redundancy analysis (5.2%, *p = *.001) (Figure 6b). The gut region was, however, the dominant explanatory variable (19.5%, *p = *.001) (Figure 6b). Globally, considering the overall time-period from day 7 to 20, ileum lumen was less diverse than the other gut niches, but given the large niche effect size, the changes in microbial community abundance under probiotic and control conditions were assessed for each gut niche separately (Figure 7). The following genera belonging to *Firmicutes* were enriched under probiotic treatment in ascending colon lumen/mucus: *Butyricicoccus*, *Clostridiales* (order), *Dorea* (OTU164), *Faecalibacterium* (OTU6, 14), *Fusicatenibacter* (OTU70), *Intestinimonas*, *Roseburia* (OTU88,98), and *Ruminococcaceae* (OTU103) (p < .05) (Figure 7, Supplementary Data Figure S8-9). Furthermore, in ascending colon/ileum lumen/mucus probiotic induced increases in *Lactobacillus* (OTU65,68), *Bifidobacterium* (OTU47,83,151), *Citrobacter* (OTU19), *Enteroccoccus* (OTU55,77), *Providencia* (OTU80), and *Veillonella* (OTU9) abundance, while *Achromobacter* (OTU60), *Akkermansia* (OTU107), and *Klebsiella* (OTU115) were significantly decreased (*p < *.05) (Figure 7, Supplementary Data Figure S8-9).

### Probiotic-treated microbiota displayed higher robustness following ETEC infection

**Figure 8.**
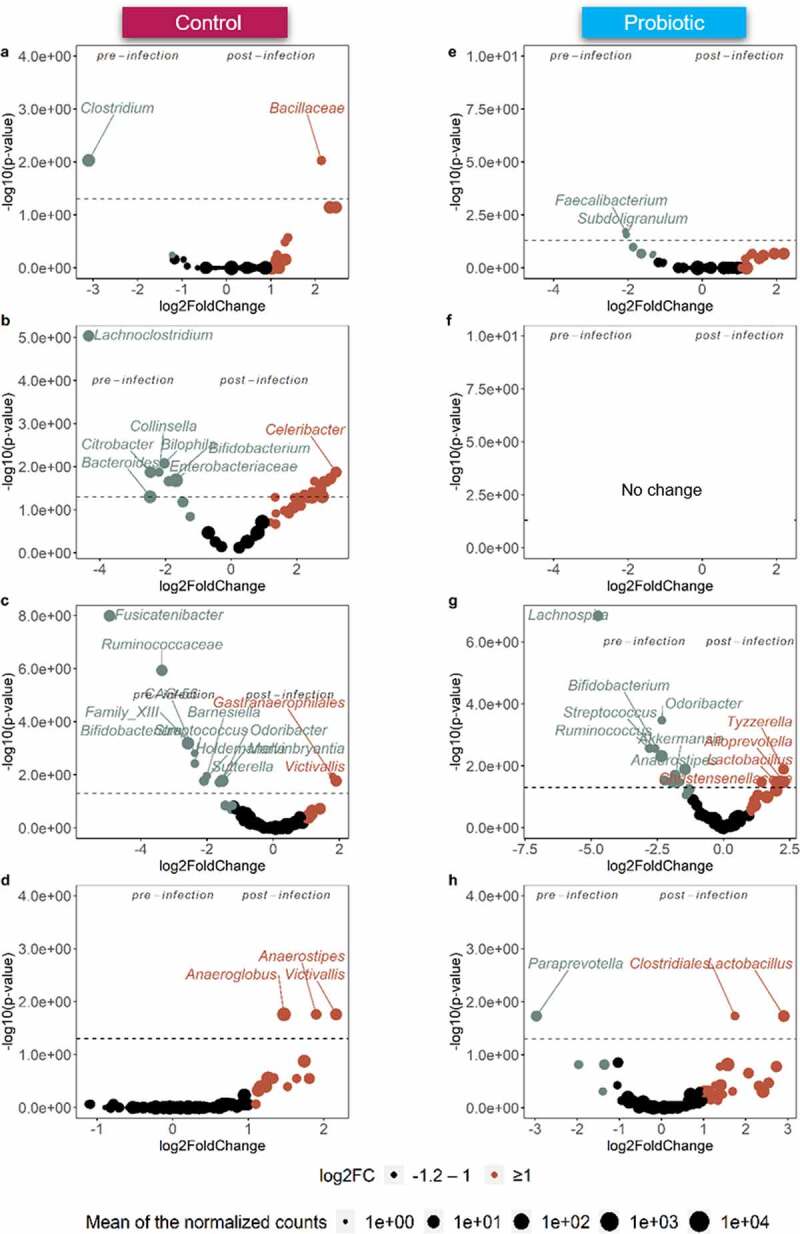
Volcano plots indicating the genera significantly enriched by ETEC infection in each gut niche of M-SHIME under control *vs* probiotic condition in (a, e) ileum lumen, (b, f) ileum mucus, (c, g) ascending colon lumen, and (d, h) ascending colon mucus. The pre-infection period (days 7–12) was compared to the post-infection period (days 13–20). A positive log_2_ fold-change indicates a reduction of the genus in post-infection phase (in brown) compared to a negative log_2_ fold-change which indicates a stimulation of the genera in the pre-infection phase (in gray). The log transformed adjusted *p*-value is displayed on the y-axis and the α = .05 significance level is indicated by a dashed line. Only the genera with an absolute log_2_ fold-change value exceeding 1.2 are represented, as determined by Deseq2 analysis

To examine the effect of ETEC infection on probiotic pre-treated *versus* control-microbiota, ETEC pre-infection (from day 7 to 12) and post-infection (from day 13 to 20) communities were compared in each gut niche (Figure 8). Globally, most of significant microbial changes (*p < *.05) following ETEC infection happened under control condition in ileum mucus (+ 10% of affected genera abundance, Figure 8b, Supplementary Data Figure S10-11) with an upsurge of rare genera such as *Mycobacterium* (OTU201), *Flavobacteriaceae* (OTU130,152,193), *Achromobacter*, *Demequina* (OTU144), *Cellvibrionales* (OTU482) replacing the pre-infection common genera, such as *Lachnoclostridium* (OTU8,57), *Enterobacteriaceae* (OTU4,19), *Collinsella* (OTU79), *Bacteroidetes* (OTU12,15), and *Bifidobacterium* (OTU35). Still under control condition, ascending colon lumen was the second most significantly affected niche (- 7.7% of affected genera abundance, Figure 8c), characterized by a decrease in *Fusicatenibacter* (OTU70), *Ruminococcaceae* (OTU103,120,209), *Bifidobacterium* (OTU47,151,376), *Clostridium family XIII* (OTU94), and *Sutterella* (Figure 8c, Supplementary Data Figure S10-11), and an enrichment of *Gastranaerophilales* (OTU248) and *Victivallis*. Remarkably, the probiotic pre-treated microbiota did not show important alteration in ileum lumen, mucus, and ascending colon mucus following ETEC infection (Figure 8(e-h)). The largest changes appeared in ascending colon lumen, with a drop (- 7.1% of affected genera abundance, Figure 8g, Supplementary Data Figure S10-11) of *Lachnospira*, *Bifidobacterium* (OTU47), *Akkermansia* (OTU107), *Ruminococcus* (OTU101), *Streptococcus*, *Odoribacter*, and *Fournierella*. In contrast, *Lactobacillus*, *Christensenellaceae*, *Alloprevotella* (OTU197), and *Tyzzerella* were stimulated (Figure 8g).

### Probiotic treatment stimulates SCFA production in ascending colon and enhances ethanol production in ileum

**Figure 9.**
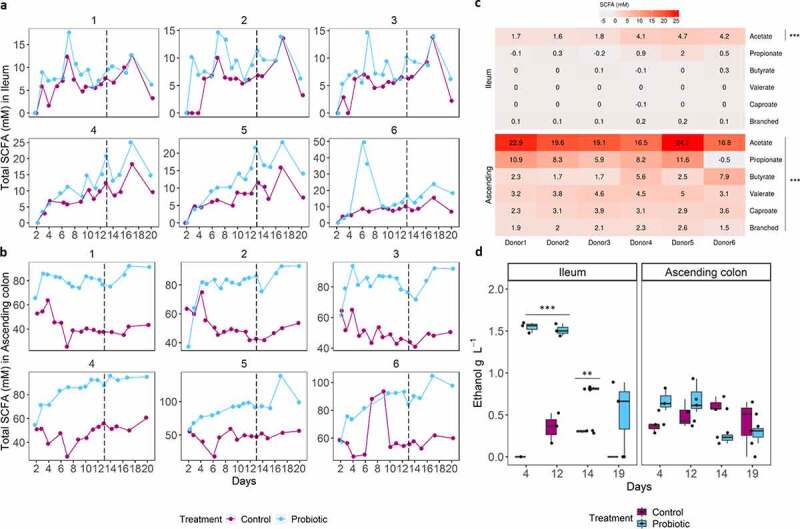
Effect of the probiotic treatment on SCFA and ethanol concentrations in ileum and ascending colon lumen of M-SHIME. (a, b) Total SCFA concentrations (in mM) are shown for each donor in the ileum (a) and (b) ascending colon lumen over the course of the 20 days lasting M-SHIME run. The vertical dashed line represents the day of ETEC infection, day 13. (c) The heatmap shows the average difference in SCFA concentrations (day 7 to 20) under the probiotic *versus* control condition. Statistically significant differences between treatments are denoted at *p* < .001 (***) as determined by pairwise wilcoxon rank sum tests with holm correction. (d) Ethanol concentrations under control and probiotic conditions measured at a few time points. Statistically significant differences between treatments are denoted at *p* < .01 (**) and *p* < .001 (***), as determined by pairwise wilcoxon rank sum tests with holm correction

The over-time impact of probiotic treatment toward microbial functionality was assessed through measurement of short-chain fatty acids (SCFA) production (Figure 9). In ileum, which displays a low metabolic activity, the effect of probiotic was limited to an acetate stimulation (Figure 9(a-c)). In ascending colon, the probiotic significantly increased total SCFA concentrations for all donors (Figure 9b) and all the measured SCFA (i.e., acetate, propionate, butyrate, caproate, valerate, and branched-fatty acids), compared to control condition (Figure 9c). The increases were most pronounced for acetate and propionate, with a 20 ± 3.1 mM and 8.5 ± 4.3 mM over donors increase, respectively, except for donor 6 where propionate concentration decreased upon treatment (Figure 9c). Valerate, caproate, and branched-fatty acids were generally low and highly similar across donors. Butyrate concentration, on the contrary, displayed a higher donor variability with a high increase noted upon probiotic addition for donors 4 and 6, compared to a small increase for donors 1, 2, 3, and 5 (Figure 9c). Lastly, ethanol, a metabolite known to be produced by *S. cerevisiae* was measured (Figure 9d). Until day 14, significantly higher concentrations were found in ileum under probiotic treatment (1.6 g L^−1^) compared to control condition (0.4 g L^−1^) (Figure 9d). No difference in ethanol concentration was found in ascending colon between both conditions.

## Discussion

**Figure 10.**
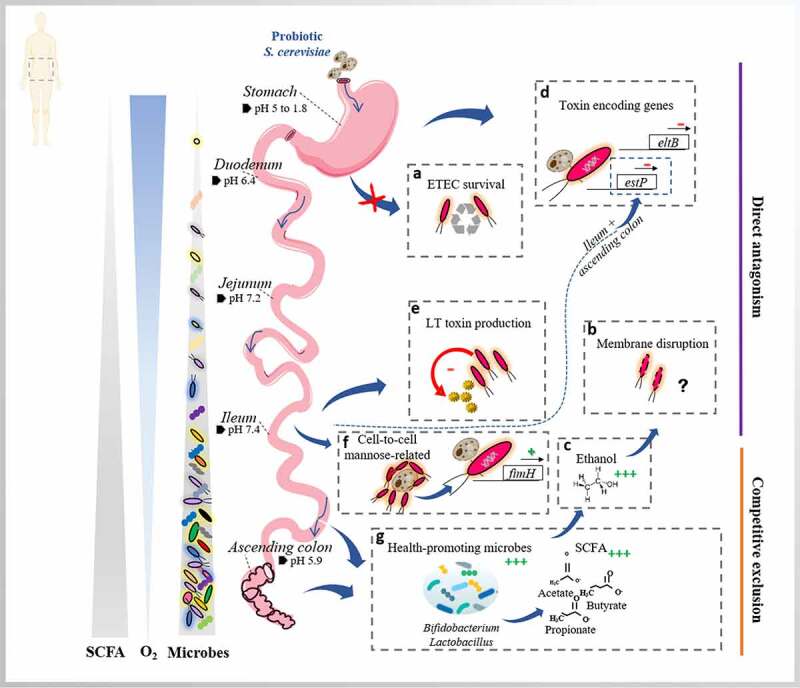
*S. cerevisiae* CNCM I-3856 regionalized mechanisms of action against ETEC H10407 in the *in vitro* human gut. On the left side, gradients of the SCFA, O_2_ and gut microbe concentrations are represented along the gastrointestinal tract as well as the region-specific pHs. On the right side, the main mechanisms of action of *S. cerevisiae* against ETEC H10407 are displayed and classified into two classes, i.e. direct antagonism and competitive exclusion

Given our recent results on the anti-infectious properties of the probiotic yeast strain *S. cerevisiae* CNCM I-3856 in simple *in vitro* models and in rodents,^17^ we set out to examine its antimicrobial properties against the human ETEC reference strain H10407 in the human upper and lower gastrointestinal tract, as simulated by the dynamic systems TIM-1 and M-SHIME, respectively. In the context of enteric diseases, the mechanisms of action of probiotics are globally classified as direct antagonism, competitive exclusion, and immunomodulatory properties.^5,21^ In the present study, we explored these mechanisms in detail (Figure 10), except for the latter since intestinal epithelial cells cannot be directly integrated into *in vitro* digestive environments.

With respect to direct antagonism, the co-administration of the probiotic with ETEC (TIM-1), as well as its continuous administration over the course of 18-days fermentation (M-SHIME) did not inhibit ETEC survival along the different *in vitro* digestive environments (Figure 10a). This lack of effect on enteric pathogen survival in a human simulated colon was consistent with another study on enterohemorrhagic *E. coli*, using the same probiotic strain.^20^ To gain further insight, the viability of the pathogen under probiotic treatment was assessed by flow cytometry (TIM-1) and PMA-qPCR (M-SHIME). The probiotic treatment seemed to contribute to the disruption of ETEC membrane integrity, as evidenced by the increase of dead and reversible damaged ETEC cells in gastric and ileal effluents of TIM-1 (Figure 10b). In the microbiota-colonized ileum and ascending colon compartments of M-SHIME, a high inter-individual variability between the six donors precluded clear-cut conclusion. The only marked effect was seen for donors 3 and 5 in the ileum, and 5–6 in the ascending colon, through the increase of dead/damaged ETEC cells number under the probiotic treatment. Interestingly, this corresponded with a particularly high concentration of ethanol (1.5 g L^−1^) observed for these donors in this specific ileal niche. We therefore hypothesize that ethanol production by the probiotic *S. cerevisiae* might contribute to the disruption of ETEC membrane integrity, as already suggested for EHEC in TIM-1 system and in other studies^18,22,23^ (Figure 10c). To further elucidate the probiotic mechanism of action, membrane potential and intracellular pH of ETEC were measured in gastric and ileal effluents of TIM-1. A modest depolarization of ETEC membrane potential was found under the probiotic condition, except at 60 min in the ileal effluents with a sharp depolarization. We hypothesize that ETEC membrane depolarization might be linked to the increased ethanol production under the probiotic treatment. In support of this hypothesis, Gyurova and Zhivkov^23^ have shown a linear decrease of the *E. coli* K12 polarization upon increasing ethanol concentrations (0 to 20 vol. %) in standard Luria Bertani (LB) medium. Another hypothesis explaining the profound ETEC membrane depolarization at early time-point in the ileal effluents might be associated with the high concentration of bile acids and the likely capacity of *S. cerevisiae* to deconjugate the bile salts, as previously found.^24^ Indeed, unconjugated bile acids are generally more hydrophobic than the corresponding conjugated forms, and therefore better solubilize membranes.^25^ With respect to pH measurements, probiotics can exert antimicrobial effects by reducing the pH of luminal microenvironment.^26^ However, in our study this feature could not be verified as the pH was controlled along the *in vitro* gastrointestinal tract. In accordance, no change in ETEC intracellular pH was found between the control and probiotic condition.

Next, probiotics can exert direct antagonistic properties through the modulation of virulence gene expression. The spatial-temporal virulence expression profile of ETEC H10407 was thus investigated under probiotic treatment in both *in vitro* simulators of the human gastrointestinal tract. We found that the probiotic co-administration triggered profound changes in ETEC virulence in gastric and ileal effluents of TIM-1. In M-SHIME, overall effects of the continuous probiotic administration were less apparent, probably due to the complex microbial background, the inherent variability across individuals and the lack of a dynamic sampling pattern to follow-up expression in a time-revolved manner. Contrary to a marked expression in ileal phase of the TIM-1, the probiotic yeast treatment tends to repress the ST enterotoxin encoding gene (*estP*) at early time-points in gastric effluents (TIM-1), and between 5 to 20 hours post-infection in ascending colon (M-SHIME) (Figure 10d). This observation is in line with two previous studies demonstrating the reduction of *estP* gene expression in ETEC-infected piglets receiving a *Lactobacillus reuteri* probiotic treatment.^27,28^ In our study, though not significant, the *eltB* gene encoding for the LT toxin tends to be repressed under the probiotic treatment, compared to the control (Figure 10d). Our ETEC strain H10407 carries the LT enterotoxin variant 1 (LT1). The probiotic treatment induced a decrease in LT1 production, significantly at some time-points in both TIM-1 and M-SHIME ileum, though inter-individual variability persisted (Figure 10e). These outcomes validate our previous observations in culture medium^17^ and are particularly relevant since enterotoxin secretion triggers the diarrheal symptoms. The underlying mechanisms of these inhibitory properties at LT gene expression and protein level remain, however, unidentified. Some speculations were made in our previous study.^17^ Other strains carrying different LT enterotoxin variants, such as the variant LT2 displayed 5-fold more toxin production.^29^ It would be interesting to assess the probiotic treatment response according to different LT variants.

The genes encoding for adhesins (*tia, fimH*, and *cfa/Ib*) were globally repressed in the stomach by the probiotic supplementation. However, in the main known site of action of ETEC, the ileum, a reverse trend was found in the TIM-1 with a significant over-expression of the genes. Said over-expression was most profound for the *fimH* gene, encoding for type 1 pili, in ileal effluents. This could be associated to mannose related interactions between ETEC and yeast cells, as previously observed in culture medium.^17^ More in particular, ETEC, through the expression of type 1 pili and its FimH subunit protein can bind to mannan moieties on the surface of yeast cells^30,31^ (Figure 10f). Even though intestinal epithelial cells cannot be directly integrated into our *in vitro* models, we assume that at the protein level, the FimH interaction with mannose residues at the surface of probiotic yeasts may potentially play a role as targets for the competitive exclusion of ETEC at mannose-containing receptors on the intestinal epithelial surface. The addition of soluble mannose in culture medium interrupted FimH-mediated ETEC adhesion to intestinal epithelium, as reported in the work of Sheikh et al.^31^ using a subclone of Caco-2 cell line. In contrast, the *fimH* gene was either basally expressed or repressed in both control and treated conditions in M-SHIME ileum and ascending colon, indicating a possible modulatory role of the gut microbiota in *fimH* gene expression. No studies have yet explored the effect of probiotic treatment on ETEC adhesin gene expression profiles even more under human simulated digestive conditions.

Finally, competitive exclusion mechanisms by *S. cerevisiae* were explored. Strategies adopted by probiotics to make the gastrointestinal microenvironment less hospitable for pathogens are broad and include the modulation of gut microbiota, the improvement of epithelium barrier function, the interference with pathogen binding or translocation, and the enhanced production of defense-associated factors, such as mucins.^5,21,32^ In the present study, we only explored the effect of *S. cerevisiae* on the gut microbiota and its metabolic activity.

We first found that the 18-days treatment with the probiotic *S. cerevisiae* positively affected the structure of the human gut ecosystem simulated in the M-SHIME, compared to the control condition. The induced changes were moreover consistent across gut regions (i.e., ileum lumen, ileum mucus, ascending colon lumen and ascending colon mucus) and donors, with the largest effects being found in the ascending colon. Generally, the community shift induced by probiotic treatment was marked by an upsurge in the abundance of *Lactobacillus* and *Bifidobacterium* in all gut microbial niches. *Lactobacillus* and *Bifidobacterium* are considered health promoting bacteria^32,33^ (Figure 10g) which might augment the defense against harmful bacteria, such as ETEC, by creating an acidic environment, synthesizing exopolysaccharides and bacteriocins, increasing antioxidant activity, producing mediators that may involve the perturbation of quorum sensing, or activating and enhancing local cell-mediated immunity against certain enteric pathogens.^32^ Other enriched genera related with health were promoted under probiotic treatment, i.e., *Veillonella dispar* in all gut niches, and *Ruminococcaceae, Ruminiclostridium, Faecalibacterium prausnitzii, Fusobacterium, Fusicatenibacter saccharivorans, Intestinimonas* in ascending colon lumen and mucus.^28,34,35^ *Enterococcus* and *Providencia* were also promoted by probiotic treatment. While some members of these pathobionts might promote disease in disturbed conditions, most of them normally act as commensals, and are even used in probiotic products (e.g., *Enterococcus faecalis*).^36^ Under non-treated control condition, a bloom of opportunistic pathogenic species appeared following ETEC challenge, such as *Klebsiella variicola, Achromobacter xylosoxidans*, and *Bilophila wadsworthia*. Those genera have been linked to intra-abdominal infections and chronic metabolic disorders.^37,38^

The observed changes in microbial composition under the probiotic treatment correlated with significant changes in metabolic activity, predominantly occurring in ascending colon. At this site, an overall increase of SCFA concentrations was found, likely resulting from the fermentation of yeast cell wall material that can serve as a substrate for other microbes. This increase was dominated by acetate, the two health markers propionate and butyrate^39^ followed by caproate and valerate (Figure 10g). Genera such as *Faecalibacterium, Butyricicoccus*, and *Roseburia* may have contributed to the butyrate production,^40^ while *Bifidobacterium, Veillonella*, and *S. cerevisiae* itself are often associated with acetate production.^41^ The unusual increase in caproate levels could be linked to the stimulation of genera like *Prevotellaceae* and *Dorea*, as described by Tap et al.^42^ Alternatively, caproate may result from the elongation of n-butyric acid by using ethanol,^43^ abundantly produced in the ileum by *S. cerevisiae* itself but also by species members stimulated under the probiotic treatment such as *Bifidobacterium longum, Enterococcus faecalis*, and *Lactobacillus fermentum*.^44^ Similar results were reported for the yeast *S. boulardii*, where it was suggested that α-mannan utilization by *Bacteroides thetaiotaomicron* stimulated butyrate production by other species.^45,46^

Next, we explored the capacity of the probiotic treatment to counteract the microbiota changes associated with ETEC infection by discriminating the pre- and post-infection periods. The probiotic treatment stabilized the relative abundance of the dominant taxa in all microbial gut niches, whereas larger detrimental changes were found in ileum mucus and ascending colon luminal communities under control conditions. Those changes were characterized by a bloom of opportunistic pathogens like *Achromobacter*,^37^ *Mycobacterium*^47^ and *Cloacibacillus*^48^ as well as the decrease of microbes with potential health benefits such as *Bifidobacterium*,^33^ *Fusicatenibacter*^49^ and *Collinsella*^50^ in the post-ETEC infection stage. This is in line with evidence suggesting that ETEC associated traveler’s diarrhea might trigger a chronic functional gastrointestinal disorder named post-infectious irritable bowel syndrome (IBS).^51,52^ No microbial marker is so far attributed to the post-infectious IBS, but decreased abundance of *Bifidobacterium* and *Lactobacillus* members as well as the actinobacterium *Collinsella aerofaciens* have been reported to promote symptoms of post-infectious IBS and diarrhea.^50–55^ While more research is required, these preliminary results indicate that the probiotic *S. cerevisiae* might aid in the prevention of the development of post-infectious IBS. Intriguingly, the synergistic effect of the probiotic treatment and ETEC infection leads to the decrease of *Akkermansia muciniphila* in the ascending colon lumen and mucus compared with the non-treated control condition. *Akkermansia spp* has been inversely associated with obesity, diabetes, inflammation, and metabolic disorders, displaying therefore promising potential as a next-generation probiotic.^56^ However, contrasting results regarding the influence of *Akkermansia spp* on gut health exist. Recently, enrichment of *Akkermansia muciniphila* was associated with neurodegenerative diseases (e.g., Alzheimer’s disease, Parkinson’s disease),^57^ and arthritis.^58^ It is important to point out that multiple strains belonging to the same *Akkermansia* species can co-exist in the gut and the modulation of host-response can be strain-specific.

## Conclusion

This study provided new insights on the use of the probiotic yeast *S. cerevisiae* CNCM I-3856 with multi-targeted actions against ETEC H10407 infection in humans. By combining the two most complete *in vitro* models of the human upper (TIM-1) and lower (M-SHIME) gastrointestinal tract in a spatiotemporal research approach we have demonstrated that the probiotic acted: (i) by suppressing ETEC virulence gene expression and LT enterotoxin production, (ii) by promoting the temporary disruption of ETEC membrane integrity through the increase of reversible damaged ETEC cells without affecting its survival, (iii) by stimulating the growth of beneficial microbes such as *Bifidobacterium* and *Lactobacillus* in the different gut microbial niches as well as, (iv) stimulating metabolic SCFA and ethanol production. Altogether, we have gathered mechanistic evidence demonstrating the probiotic activity in a host-independent way, regardless of the inherent variability across microbiome-specific individuals. Such interpersonal differences in the probiotic response fall within the emerging concept of individuality for personalized nutrition and/or medicine.^59^ Our encouraging results merit further investigations, including host factors, by coupling the digestive systems with human intestinal cells or organoids.

## Methods

### Strains and growth conditions

The prototypical ETEC strain H10407 serotype O78:H11:K80 (LT^+^, ST^+^, CFA/I^+^) was used in this study.^60^ ETEC was routinely grown under agitation (37°C, 125 rpm, overnight) in LB broth (BD Difco, Waltham, USA) until OD_600nm_ = 0.6 (stationary phase). The probiotic yeast strain *S. cerevisiae* CNCM I-3856 was provided by Gnosis from Lesaffre (a Business Unit of Lesaffre Group, France). This strain is a proprietary and patented strain of Lesaffre, registered in the French National Collection of Microorganisms (CNCM). *S. cerevisiae* specie was determined using phenotypic (API®ID32C, Biomerieux SAS, Marcy l’Etoile, France) and genotypic methods (genetic amplification and sequencing of 26S DNA).^61,62^ Moreover, the strain I-3856 has been characterized by polymerase-chain reaction (PCR) interdelta type techniques^63^ and complete genome sequencing. Prior to an experiment, the probiotic was resuspended in sterile saline water and homogenized with an ultra-turrax yellow line (IKA, Rawang, Malaysia).

### TIM-1 gastrointestinal system

The experimental set-up of TIM-1 was previously described.^64^ The TIM-1 system was programmed based on *in vivo* data to simulate the physicochemical digestive conditions from the stomach to the ileum, encountered in a healthy adult.^65^ The system does not include microbial ecosystem, contrary to the M-SHIME (section below). Three conditions were tested in TIM-1 (Figure 1a): (i) control experiment consisted of feeding the stomach compartment with mineral water (200 mL) experimentally contaminated with ETEC (7.5 log_10_ CFU mL^−1^); (ii) probiotic control condition consisted in the administration of the yeast *S. cerevisiae* alone (7.5 log_10_ CFU mL^−1^); and (iii) probiotic treatment condition consisted in the co-administration of ETEC (7.5 log_10_ CFU mL^−1^) and the probiotic yeast (7.5 log_10_ CFU mL^−1^). Digestions were run in quadruplicate. The initial bacterial suspension (T0) was collected, and samples were regularly taken during *in vitro* digestions from each digestive compartment (stomach, duodenum, jejunum, and ileum). Gastric effluents were collected on ice and pooled after 0–10, 10–20, 20–40 and 40–60 min of gastric digestion, while ileal effluents were taken on an hourly basis for a 5-h period. Samples collected for plating and flow cytometry were processed immediately. Pellets for DNA-extraction were stored at −20°C, while RNA samples were resuspended in 500 µL RNA*later*® (Thermo Fisher Scientific, San José, US) prior to storage at −80°C. Supernatants were stored at −20°C for measurement of the LT enterotoxin by ELISA assays.

### M-SHIME fermentation system

The M-SHIME® consists of a series of connected double-jacketed reactors (Pierreglas, Vilvoorde, Belgium) mimicking conditions of the upper and lower part of the human gastrointestinal tract, operated in a semi-continuous mode to mimic gastrointestinal transit.^66,67^ Three successive compartments simulating the combined stomach/duodenum-jejunum, the ileum and the ascending colon were run in parallel for a probiotic *vs* control condition (Figure 1b). Only ileum and ascending colon were inoculated with fecal microbiota. To capture interindividual variability in ETEC behavior and probiotic effects, six healthy adults were inoculated the M-SHIME (3 women and 3 men aged between 25 and 36 years old, including Belgian, African, Turkish, and French origins with two vegetarians). Fresh fecal samples were collected in sterile airtight containers comprising anaerogen bags (BD GasPak™, Erembodegem, Belgium). Consent for fecal collection was obtained under registration number BE670201836318 (Gent University). A 20% (w/v) fecal slurry was prepared as previously described.^67^ All vessels were flushed with N_2_ immediately after inoculation to generate anaerobic conditions. In addition, the mucosal environment was mimicked in both ileum and ascending colon compartments, through the incorporation of microcosms (AnoxKaldnes K1 carrier, Lund, Sweden) coated with type III porcine mucin-agar (Sigma͐–aldrich, St. Louis, US), instead of type II mucin, as described by Van den Abbeele et al.^68^ Functioning of the system, mucin carrier replacement and media composition have been presented in Roussel et al.^65^

Microbiota derived from the six human donors were tested under both control and probiotic conditions (Figure 1b), as following: (i) the probiotic treatment consisted of the introduction of the yeast *S. cerevisiae* CNCM I-3856 resuspended in 30 mL sterile water (7.5 log_10_ CFU mL^−1^) in the SHIME stomach, twice a day (9 a.m and 5 p.m) during 18-days from day 2 to 20; while under (ii) control condition, a sham treatment with 30 mL sterile water was performed during day 2 to 20. Prior to ETEC challenge, both conditions control and probiotic treatment include a stabilization period of 12 days that is typically applied in the M-SHIME to ensure ecological stability as previously investigated.^69^ ETEC challenge was therefore tested under both conditions, the pre- (negative ETEC control) *vs* post-ETEC (positive) infection were discriminated as following: (i) the days 7 to 12 were kept as pre-infection period (bioreactors were stable from day 7 based on SCFA measurements); while (ii) at day 13, both control and probiotic treated systems were challenged with ETEC by inoculation of 7.5 log_10_ CFU mL^−1^ in SHIME ileum vessels. The days 13 to 20 were kept as post-infectious period. Prior to the challenge at day 13, ETEC and *S. cerevisiae* were pre-digested 3 h under batch conditions, to reproduce the gastro-jejunal digestion of a glass of mineral water, where physicochemical conditions were close to those found in TIM-1 (without nutritional medium, under aerobic conditions).^65^ SHIME suspensions from ileum and ascending colon vessels were sampled every two days for SCFA analysis and ethanol production. DNA, RNA samples, and supernatants for ELISA measurement were collected at different hours from day 13, and until 29 h post-infection and stored as previously explained for TIM-1 samples. Mucus samples were obtained every 2–3 days.^70^ 250 mg mucus was aliquoted and stored at −20°C before DNA extraction.

### DNA extraction

Total DNA from TIM-1 and M-SHIME experiments was extracted according to Geirnaert et al.^70^ DNA samples were stored at −20°C and the quality was analyzed by gel electrophoresis (1.2% w/v agarose) (Life technologies, Madrid, Spain). DNA extracts were diluted 1:10 in 1X TE buffer (Tris and EDTA) for ETEC qPCR quantification.

### ETEC and probiotic quantification

The number of cultivable ETEC in each digestive compartment of TIM-1 was determined by direct plating onto LB agar (overnight incubation at 37°C). Total ETEC bacteria and the viable ones were also measured after DNA extraction of digestive samples. DNA were pelleted in duplicate (6,339 *× g*, 10 min, 4°C). One aliquot was stained with 50 μM propidium monoazide (PMA, Interchim, Montluçon, France).^65^The qPCR procedure was performed using the StepOnePlus Real-Time PCR system (Applied Biosystems) with *16S Enterobacteriaceae* primers for TIM-1 and *gspD* primers for M-SHIME, as previously described.^65^ Probiotic yeasts were plated onto Sabouraud agar (BD Difco, Waltham, USA), supplemented with chloramphenicol (50 mg L^−1^) (Sigma, St Louis, USA) and incubated at 30°C for 48 h. All statistical analyses were performed in R studio, version 3.6.1 (R Core Team, 2019), using PMCMR package, version 4.3. All formal hypothesis tests were conducted on the 5% significance level (*p ≤ *0.05). Non-parametric tests were performed to assess the pairwise comparison of: (i) the probiotic *S. cerevisiae* survival in comparison with the transit marker given by the TIM-1 system and (ii) ETEC survival under control *versus* probiotic conditions in the TIM-1 and M-SHIME using Mann–Whitney (Wilcoxon- Rank-Sum) test with Holm correction.

### Flow cytometry analysis

Those analysis were conducted only in the TIM-1 system. Five mL of gastric or ileal effluents from TIM-1 were centrifuged (9,000 *× g*, 5 min, 20°C). Pellets were resuspended into PBS 1X at pH 7.3 to obtain approximately 6 log_10_ cells mL^−1^. Flow cytometry analysis was performed on a CyFlow SL cytometer and data were collected with FlowMax software version 2.3 (Sysmex Partec, Görlitz, Germany).

*Live/Dead ETEC quantification*. Bacteria were stained using the Live/Dead BacLight^TM^ Kit (L34856 Molecular Probes, Waltham, US), consisting of the green-fluorescent DNA stain SYTO9 labeling all bacteria and the red-fluorescent propidium iodide only penetrating and staining cells with damaged membranes, according to the protocol previously described.^64^

*ETEC membrane potential*. The probe 3,3ʹ-diethyloxacarbocyanine iodide (DiOC_2_(3) and the proton ionophore carbonyl cyanide m-chlorophenyl hydrazone (CCCP) were used according to the manufacturer’s instructions (BacLightTM Kit B34950 Thermo Fisher Scientific, Waltham, USA). Briefly, DiOC_2_(3) at low concentration exhibits green fluorescence in all bacterial cells.^71^ However, the dye becomes more concentrated in healthy cells that are maintaining a membrane potential, causing the dye to self-associate and the fluorescence emission to shift to red. The CCCP was used as a control to eradicate the proton gradient, eliminating thus the bacterial membrane potential (depolarized membranes).^71^ Analysis was performed using fluorescence emission ratio detection for bacteria incubated with 30 µM DiOC_2_(3) for 30 minutes at room temperature in the dark in either the presence or absence of 5 µM CCCP. Based on the fluorescence intensity (FI) ratio (sample/control), membranes are depolarized (FI ratio is approaching 1) and polarized (FI ratio exceeding 1.2).

### Transcriptional analysis by quantitative real-time qPCR and LT-monosialoganglioside (GM1) ELISA

Total RNA was extracted from TIM-1 and SHIME digestive samples using the TRIzol® method (Invitrogen, Thermo Fisher Scientific, Waltham, USA) according to the author recommendation.^65^ DNAse treatment and RNA quality control were performed respectively, according to the manufacturer’s recommendation (TURBO DNA-free^TM^, Invitrogen, Thermo Fisher Scientific, Waltham, USA) and Roussel et al.^65^ RT-qPCR was used to analyze the expression of seven virulence genes encoding for enterotoxins (*eltB* and *estP*), enterotoxin release (*leoA* and *tolC*), and adhesins (*cfa/Ib, tia, fimH*).^65^ Non-parametric test was used to compare the log_2_ fold change in gene expression under control *versus* probiotic conditions using Nemenyi post-hoc test conducted following significant results for the Friedman test.

LT enterotoxins were measured in supernatants collected from TIM-1 and M-SHIME.^72^ Optical density was read at 450 nm using the multiscan Tecan Infinite® 200 PRO. LT toxin concentrations were expressed in pg mL^−1^. Statistical comparison of the LT enterotoxin production under control *versus* probiotic condition was made with Fisher post-hoc test following significant results for the Friedman test.

### Microbial community analysis

Following SHIME experiments, next-generation *16S* rRNA gene amplicon sequencing of the V3-V4 region (341 F-785 R) was performed by LGC Genomics (Teddington, Middlesex, UK), on an Illumina MiSeq platform with Illumina V3 chemistry using the 600-cycle reagent kit (Illumina, Hayward, US).^73^ The sequence data have been submitted to the NCBI (National Center for Biotechnology Information) database under accession number PRJNA562529.

*Bioinformatics analysis*. The mothur software package (version 1.40.5) and guidelines were used to process the Illumina amplicon sequencing data generated by LGC Genomics.^74^ OTUs were defined as a collection of sequences with a length between 400 and 428 nucleotides that are found to be more than 97% similar to one another in the V3-V4 region of their *16S* rRNA gene after OptiClust clustering.^75–77^ Taxonomy was assigned using the RDP database.^77,78^ The shared file, containing the number of reads observed for each OTU in each sample, was loaded into R version 3.6.1 (R Core Team, 2019). Singletons were removed^79^ and UCHIME was applied to remove chimera.^80^ For the most abundant OTUs the sequences retrieved from the 3% dissimilarity level FASTA file, obtained in mothur, were classified through the RDP web interface using the RDP SeqMatch tool (restricting the search to type strains with only near-full-length good quality sequences) and blasted in NCBI against the *16S* rRNA gene sequences, selecting only type material, with optimization of the BLAST algorithm for highly similar sequences.^78,81^ Although a level of uncertainty is introduced by classification to the species level based on short 300 bp reads, the best hit returned by both databases is used to refer to interesting OTUs in the results section of this article. In case of inconsistencies between the RDP SeqMatch tool and NCBI BLAST, no species level classification was mentioned.

*Statistical analysis of amplicon data*. All statistical analyses were performed in R, version 3.6.1 (R Core Team, 2019). All formal hypothesis tests were conducted on the 5% significance level. To visualize differences in microbial community composition between treatments, donors, periods (e.g., pre- and post-infection) and gut regions (e.g., ileum lumen, ileum mucus, ascending lumen, and ascending mucus), ordination and clustering techniques were applied. For these purposes, the shared file was further processed to remove OTUs with too low abundance according to the arbitrary cutoffs described by McMurdie and Holmes (2014).^79^ An OTU should be observed in 5% of the samples and read counts should exceed 0.5 times the number of samples.^79^ Rarefaction curves were constructed to assure that the samples were sequenced in sufficient depth.^82^ To deal with differences in sampling depth, proportional data transformed on the common scale to the lowest number of reads was used.^76^ The influence of the treatments, gut regions, donors, and periods was determined by applying a distance-based redundancy analysis (db RDA) using the abundance-based jaccard distance as a response variable (vegan 2.5–6)^82,83^ and visualized with ggplot2 3.2.1. The factor treatment (control, probiotic) was used as a constraint with the effect of gut regions (ileum lumen, ileum mucus, ascending colon lumen, ascending colon mucus), donors (1 to 6) and periods (pre- and post-infection) being partially out. Interpretation of the results was preceded by a permutation test of the RDA results to confirm that a linear relationship exists between the response data and the exploratory variables. The constrained fraction of the variance explained by the exploratory variables was adjusted by applying Ezekiel’s formula.^84^ This procedure was repeated on species and genus levels. On the genus level, weighed averages of genera abundances were a *posteriori* added to the ordination plot using the wascores function in vegan.^82^ To confirm the trends, observed data was clustered by means of an Unweighted Pair-Grouped Method using arithmetic Averages (UPGMA) clustering method (cluster 2.1.0).^85^ The significance of the observed group separation between gut region, donor, and period in the PCoA was assessed with a Permutational Multivariate Analysis of Variance (PERMANOVA) using distance matrixes (vegan 2.5–6).^82,83^ Prior to this formal hypothesis testing, the assumption of similar multivariate dispersions was evaluated.

In order to find statistically significant differences in species abundance between the treatments and/or pre- and post-infection periods, the DESeq2 package 1.26.0 was applied.^79,85^ The factors treatment, period, gut region and donor were used in the design of LRT formula. Statistical differences between the treatments and/or the pre- and post-infection periods were determined using a Wald Test.

### Metabolite production

Luminal samples from M-SHIME were diluted 1:2 with milliQ® water (Merck, Darmstadt, Germany) to a total volume of 2 mL. SCFA production was measured using capillary gas chromatography coupled to a flame ionization detector after diethyl ether extraction.^86^ SCFA concentrations were expressed in mM. After a 10-fold dilution in milli-Q sterile water, ethanol concentrations were determined using a HPLC system (Shimadzu Prominence HPLC system, Columbia, MD, US).^86^ Statistical hypothesis testing to assess the effect of ETEC infection on the metabolic activity (SCFA, ethanol) was performed by using the Kruskal–Wallis rank sum test, followed by Pairwise Wilcoxon Rank Sum Test with Holm correction for multiple testing.

## Supplementary Material

Supplemental Material

## Data Availability

Raw 16S rRNA gene sequence data were made publicly available online through the Sequence Read Archive (SRA) portal of NCBI under accession number PRJNA562529 (https://www.ncbi.nlm.nih.gov/bioproject/PRJNA562529/).
