# Supplementary material for: Multi-targeted properties of the probiotic saccharomyces cerevisiae CNCM I-3856 against enterotoxigenic escherichia coli (ETEC) H10407 pathogenesis across human gut models
Source: Gut Microbes. 2021 Aug 25;13(1):1953246. doi: 10.1080/19490976.2021.1953246 (PMC8405159; doi:10.1080/19490976.2021.1953246)
Supplement: Supplemental Material [file KGMI_A_1953246_SM3559.zip › supplementary/Supplementary dataGutMicrobes2021.doc]

Supplementary Materials

**Multi-targeted properties of the probiotic *S. cerevisiae* CNCM I-3856 against enterotoxigenic *E. coli* (ETEC) H10407 pathogenesis across human gut models**

**Supplementary Tables**

**Supplementary Table S1. Effect of the probiotic treatment on ETEC intracellular pH (pHi) in the TIM-1 system.** The carboxyfluorescein diacetate succinimidyl ester CFDA-SE Vybrant Kit (CFDA SE cell Tracer, Kit V12883 Thermo Fisher Scientific, Waltham, USA) was used to measure ETEC pHi by flow cytometry. The fluorescence intensity from each sample (e.g. initial inoculum, T 20 min in the stomach, T 180 min in the duodenum and T 300 min in the ileum) was obtained from the calibration curves expressed according to the pHi. Extracellular pH (pHext) are also given in each digestive compartment. The table shows the mean of two independent replicates ± SD.

| **Digestive compartment** | **pHext** | **pHi control** | **pHi probiotic** |
| --- | --- | --- | --- |
| **Inoculum (T0)** | 6.5 ± 0.1 | 6.6 ± 0.1 | 6.4 ± 0.1 |
| **Stomach (T20 min)** | 2.5 ± 0.2 | 3.0 ± 0.1 | 2.8 ± 0.1 |
| **Duodenum (T180 min)** | 6.5 ± 0.2 | 5.8 ± 0.3 | 5.5 ± 0.2 |
| **Ileum (T300 min)** | 7.0 ± 0.1 | 6.1 ± 0.2 | 6.0 ± 0.1 |

**Supplementary Table S2. RDP Seqmatch and NCBI BLAST results for the most abundant species and/or species of interest in the M-SHIME.** The similarity score (Sab), as calculated by RDP, and the NCBI BLAST output for the best hit and next best hit(s) is shown. The NCBI maximal score (not shown) equalled the total score for all displayed entries. Indicated in bold, the OTU numbers for which they have been replaced by the species names for the microbial analysis. NA= Not Available.

|  |  | **RDP** | **NCBI BLAST** | | | |
| --- | --- | --- | --- | --- | --- | --- |
| **OTU** | **Species** | **Sab** | **Total score** | **Query coverage (%)** | **E-value** | **Identity (%)** |
| **1** | *Escherichia/Shigella fergusonii*  *Escherichia/Shigella flexneri*  *Shigella sonei*  *Escherichia coli*  *Escherichia vulneris* | 1.000  1.000  1.000  1.000  1.000 | 787  787  787  787  787 | 100  100  100  100  100 | 0.0  0.0  0.0  0.0  0.0 | 100  100  100  100  100 |
| **2** | *Anaerovibrio lipolyticus*  *Selenomonas bovis* | 0.771  0.752 | 638  614 | 99  100 | 0.0  1e-175 | 94  93 |
| **3** | ***Klebsiella pneumoniae***  *Klebsiella quasipneumoniae*  *Klebsiella variicola*  *Serratia liquefaciens* | **0.969**  0.969  0.964  - | **782**  776  -  776 | **100**  100  -  100 | **0.0**  0.0  -  0.0 | **99**  99  -  99 |
| **4** | *Leclercia adecarboxylata*  *Enterobacter cloacae*  *Enterobacter ludwigii*  *Pantoea agglomerans*  *Enterobacter kobei*  *Salmonella enterica* | 1.000  0.983  1.000  -  0.983  0.983 | 787  787  787  787  784  782 | 100  100  100  100  100  100 | 0.0  0.0  0.0  0.0  0.0  0.0 | 100  100  100  99  99  99 |
| **5** | ***Mitsuokella multacida***  *Mitsuokella jalaludinii*  *Selenomonas bovis* | **0.983**  0.954  0.814 | **784**  754  630 | **100**  100  99 | **0.0**  0.0  1e-180 | **99**  99  93 |
| **6** | ***Faecalibacterium prausnitzii***  *Gemmiger formicilis* | **0.982**  0.716 | **736**  569 | **100**  100 | **0.0**  7e-159 | **99**  92 |
| **7** | *Succinivibrio dextrinosolvens*  *Anaerobiospirillum succiniciproducens* | 0.919  0.646 | 688  540 | 100  100 | 0.0  2e-153 | 98  91 |
| **8** | ***Clostridium bolteae***  *Clostridium asparagiforme* | **1.000**  0.923 | **741**  986 | **100**  100 | **0.0**  0.0 | **100**  98 |
| **9** | ***Veillonella dispar***  *Veillonella tobetsuensis*  *Veillonella parvula* | **0.968**  0.949  0.929 | **776**  760  754 | **100**  100  100 | **0.0**  0.0  0.0 | **99**  99  99 |
| **10** | ***Bacteroides fragilis***  *Bacteroides ovatus* | **1.000**  - | **778**  649 | **100**  100 | **0.0**  0.0 | **100**  95 |
| **11** | *Mitsuokella jalaludinii*  *Mitsuokella multacida* | 0.983  0.937 | 782  750 | 100  100 | 0.0  0.0 | 99  98 |
| **12** | ***Bacteroides dorei***  *Bacteroides vulgatus* | **1.000**  0.951 | **778**  756 | **100**  100 | **0.0**  0.0 | **100**  99 |
| **13** | ***Pseudomonas aeruginosa***  *Pseudomonas guezennei* | **1.000**  - | **787**  776 | **100**  100 | **0.0**  0.0 | **100**  99 |
| **14** | ***Faecalibacterium prausnitzii***  *Gemmiger formicilis* | **0.918**  0.735 | **713**  592 | **100**  100 | **0.0**  7e-169 | **99**  93 |
| **15** | ***Bacteroides thetaiotaomicron***  *Bacteroides faecichinchillae*  *Bacteroides faecis* | **1.000**  0.947  0.935 | **778**  756  739 | **100**  100  100 | **0.0**  0.0  0.0 | **100**  99  98 |
| **16** | ***Bacteroides uniformis***  *Bacteroides rodentium* | **1.000**  0.906 | **778**  717 | **100**  100 | **0.0**  0.0 | **100**  97 |
| **17** | *Acidaminococcus fermentans*  *Acidaminococcus intestini* | 0.925  - | 737  671 | 100  100 | 0.0  0.0 | 98  95 |
| **18** | ***Clostridium butyricum***  *Clostridium saccharobutylicum* | **1.000**  0.941 | **741**  719 | **100**  100 | **0.0**  0.0 | **100**  99 |
| **19** | *Citrobacter freundii*  *Citrobacter brakii* | 0.990  0.971 | 782  771 | 100  100 | 0.0  0.0 | 99  99 |
| **20** | ***Acidaminococcus intestine***  *Acidaminococcus fermentans* | **1.000**  0.885 | **787**  704 | **100**  100 | **0.0**  0.0 | **100**  96 |
| **21** | ***Gemmiger formicilis***  *Subdoligranulum variabile* | **1.000**  0.959 | **741**  719 | **100**  100 | **0.0**  0.0 | **100**  99 |
| **22** | *Bacteroides timonensis*  *Bacteroides cellulosilyticus*  *Bacteroides intestinalis* | -  0.934  0.888 | 761  750  739 | 100  100  100 | 0.0  0.0  0.0 | 99  99  98 |
| **23** | ***Eubacterium rectale***  *Roseburia faecis*  *Roseburia intestinalis* | **0.949**  0.941  0.892 | **741**  713  939 | **100**  100  100 | **0.0**  0.0  0.0 | **100**  99  98 |
| **24** | ***Parabacteroides distasonis***  *Parabacteroides gordonii*  *Parabacteroides faecis* | **0.971**  0.672  0.613 | **778**  756  601 | **100**  100  100 | 0.0 2e-174; 1e-171 | **100**  93  92 |
| **25** | *Bacteroides ovatus*  *Bacteroides xylanisolvens* | 0.978  - | 773  730 | 100  100 | 0.0  0.0 | 99  98 |
| **26** | ***Phascolarctobacterium faecium***  *Phascolarctobacterium succinatutens* | **1.000**  0.734 | **787**  632 | **100**  100 | **0.0**  0.0 | **100**  93 |
| **27** | ***Clostridium perfringens***  *Eubacterium tarantellae* | **1.000**  - | **741**  691 | **100**  100 | **0.0**  0.0 | **100**  98 |
| **28** | *Mitsuokella jalaludinii*  *Mitsuokella multacida*  *Selenomonas bovis* | 0.935  0.886  - | 704  673  675 | 100  100  100 | 0.0  0.0  0.0 | 96  95  95 |
| **29** | *Bilophila wadsworthia*  *Desulfovibrio simplex* | 0.973  - | -  604 | -  100 | -  9e-173 | -  92 |
| **30** | ***Blautia faecis***  *Blautia glucerasea* | **1.000**  0.926 | **741**  726 | **100**  100 | **0.0**  0.0 | **100**  99 |
| **35** | ***Bifidobacterium longum***  *Bifidobacterium breve* | **0.985** | **750** | **100** | **0.0** | **100** |
|  |  | 0.918 | - | - | - | - |
|  |  |  |  |  |  |  |
| **40** | *Prevotella copri*  *Prevotella oulorum* | 0.851  0.751 | 717  640 | 100  100 | 0.0  0.0 | 97  94 |
| **44** | *Prevotella salivae*  *Prevotella shahii* | 0.749  0.712 | 606  - | 100  - | 2e-173  - | 93  - |
| **47** | *Bifidobacterium faecale*  *Bifidobacterium adolescentis* | 1.000  1.000 | 758  758 | 100  100 | 0.0  0.0 | 100  100 |
| **49** | ***Phascolarctobacterium succinatutens***  *Phascolarctobacterium faecium* | **0.958**  0.724 | **765**  638 | **100**  100 | **0.0**  0.0 | **99**  94 |
| **52** | ***Eubacterium ventriosum***  *Lachnospiraceae bacterium* | **0.961**  0.765 | **726**  628 | **100**  99 | **0.0**  5e-180 | **99**  95 |
| **53** | ***Marivita hallyeonensis***  *Marivita roseacus* | **0.964**  0.940 | **730**  719 | **100**  100 | **0.0**  0.0 | **99**  99 |
| **55** | ***Enterococcus faecium*** | **1.000** | **787** | **100** | **0.0** | **100** |
| **60** | *Achromobacter denitrificans*  *Achromobacter pulmonis*  *Achromobacter agilis*  *Achromobacter xylosoxidans* | 0.978  0.978  -  0.958 | 776  776  776  776 | 100  100  100  100 | 0.0  0.0  0.0  0.0 | 99  99  99  99 |
| **65** | ***Lactobacillus fermentum*** | **1.000** | **787** | **100** | **0.0** | **100** |
| **66** | ***Clostridium aldenense***  *Clostridium algidixylanolyticum*  *Lachnoclostridium pacaense* | **0.964**  0.835  - | **730**  -  708 | **100**  -  100 | **0.0**  -  0.0 | **99**  -  99 |
| **68** | ***Lactobacillus gasseri*** | **1.000** | **787** | **100** | **0.0** | **100** |
| **70** | ***Fusicatenibacter saccharivorans***  *Murimonas intestine* | **1.000**  0.856 | **741**  669 | **100**  100 | **0.0**  0.0 | **100**  97 |
| **72** | ***Synergistetes bacterium***  *Cloacibacillus porcorum* | **1.000**  0.916 | **-**  730 | **-**  100 | **-**  0.0 | **-**  99 |
| **77** | ***Enterococcus faecalis*** | **1.000** | **787** | **100** | **0.0** | **100** |
| **79** | ***Collinsella aerofaciens***  *Collinsella bouchesdurhonensis* | **1.000**  - | **743**  675 | **100**  100 | **0.0**  0.0 | **100**  97 |
| **80** | ***Providencia vermicola*** | **1.000** | **743** | **100** | **0.0** | **100** |
| **81** | ***Paraprevotella clara***  *Paraprevotella xylaniphila* | **0.969**  0.685 | **765**  604 | **100**  100 | **0.0**  0.0 | **99**  93 |
| **83** | ***Bifidobacterium bifidum*** | **1.000** | **787** | **100** | **0.0** | **100** |
| **85** | ***Sutterella wadsworthensis***  *Sutterella stercoricanis* | **1.000**  - | **787**  654 | **100**  100 | **0.0**  0.0 | **100**  94 |
| **88** | *Roseburia sp.*  *Eubacterium eligens* | 1.000  - | -  625 | -  100 | -  6e-179 | -  95 |
| **90** | ***Clostridium scindens***  *Dorea longicatena* | **1.000**  - | **741**  669 | **100**  100 | **0.0**  0.0 | **100**  97 |
| **91** | *Clostridium chromiireducens*  *Clostridium butyricum*  *Clostridium saccharobutylicum* | -  0.982  - | 725  719  708 | 100  100  100 | 0.0  0.0  0.0 | 99  99  99 |
| **94** | *Clostridium celerecrescence*  *Clostridium xylanolyticum* | 0.860  0.857 | 686  - | 100  - | 0.0  - | 98  - |
| **98** | ***Roseburia hominis*** | **1.000** | **743** | **100** | **0.0** | **100** |
| **102** | *Clostridium colinum*  *Clostridium piliforme* | 0.830  0.828 | 658  - | 100  - | 0.0  - | 96  - |
| **103** | *Ruminococcaceae bacterium*  *Oscillibacter ruminantium* | 1.000  - | -  573 | -  100 | -  2e-163 | -  92 |
| **104** | ***Bacteroides caccae***  *Bacteroides faecis* | **1.000**  0.850 | **778**  723 | **100**  100 | **0.0**  0.0 | **100**  98 |
| **107** | ***Akkermansia muciniphila*** | **1.000** | **752** | **100** | **0.0** | **100** |
| **115** | ***Klebsiella variicola***  *Klebsiella pneumonia* | **0.983**  0.959 | **782**  776 | **100**  100 | **0.0**  0.0 | **99**  99 |
| **117** | ***Clostridium tertium***  *Clostridium chauvoei* | **1.000**  0.985 | **741**  736 | **100**  100 | **0.0**  0.0 | **100**  99 |
| **120** | *Oscillibacter valericigenes*  *Oscillibacter ruminantium* | 0.778  0.745 | 617  606 | 100  100 | 1e-176  2e-173 | 94  94 |
| **124** | ***Blautia luti***  *Blautia stercoris* | **0.987**  0.926 | **736**  691 | **100**  100 | **0.0**  0.0 | **99**  98 |
| **127** | ***Alistipes shahii***  *Alistipes finegoldi* | **0.968**  0.924 | **773**  739 | **100**  100 | **0.0**  0.0 | **99**  98 |
| **131** | ***Blautia obeum***  *Blautia wexlerae* | **1.000**  0.916 | **737**  686 | **100**  100 | **0.0**  0.0 | **99**  98 |
| **151** | ***Bifidobacterium angulatum***  *Bifidobacterium merycicum* | **1.000**  0.974 | **754**  737 | **100**  99 | **0.0**  0.0 | **100**  99 |
| **158** | ***Eisenbergiella tayi*** | **1.000** | **741** | **100** | **0.0** | **100** |
| **160** | ***Victivallis vadensis***  *Caloramator proteoclasticus* | **1.000**  - | **743**  331 | **100**  99 | **0.0**  2e-90 | **100**  82 |
| **163** | ***Ruminococcus faecis***  *Ruminococcus torques* | **1.000**  0.985 | **741**  682 | **100**  100 | **0.0**  0.0 | **100**  97 |
| **164** | ***Dorea formicigenerans*** | **1.000** | **741** | **100** | **0.0** | **100** |
| **166** | ***Bacteroides eggerthii***  *Bacteroides uniformis* | **1.000**  0.857 | **778**  712 | **100**  100 | **0.0**  0.0 | **100**  97 |
| **175** | *Blautia coccoides*  *Muricome intestine*  *Clostridium oroticum* | 0.867  0.824  0.810 | 680  675  669 | 100  100  100 | 0.0  0.0  0.0 | 97  97  97 |
| **201** | *Mycobacterium moriokaense*  *Mycobacterium grossiae*  *Mycobacterium aquaticum* | 0.915  -  - | 701  701  701 | 100  100  100 | 0.0  0.0  0.0 | 98  98  98 |

**Supplementary Figures**

**Supplementary Figure S1. Phylum level microbial community composition of the luminal gut environment in the M-SHIME.** Ileum (a) and ascending colon (b) environments under control and probiotic conditions from six different donors over the course of 20 days fermentation. ETEC infection started at day 13. Relative abundances were determined by amplicon sequencing.

**Supplementary Figure S2. Phylum level microbial community composition of the mucosal gut environment in the M-SHIME.** Ileum (a) and ascending colon (b) environments under control and probiotic conditions from six different donors over the course of 20 days fermentation. ETEC infection started at day 13. Relative abundances were determined by amplicon sequencing.

**Supplementary Figure S3. Genus level microbial community composition of the luminal gut environment in the M-SHIME.** Ileum (a) and ascending colon (b) environments under control and probiotic conditions from six different donors over the course of 20 days fermentation. ETEC infection started at day 13. Relative abundances were determined by amplicon sequencing.

**Supplementary Figure S4. Genus level microbial community composition of the mucosal gut environment in the M-SHIME.** Ileum (a) and ascending colon (b) environments under control and probiotic conditions from six different donors over the course of 20 days fermentation. ETEC infection started at day 13. Relative abundances were determined by amplicon sequencing.

**Supplementary Figure S5. Species level microbial community composition of the luminal gut environment in the M-SHIME.** Ileum (a) and ascending colon (b) environments under control and probiotic conditions from six different donors over the course of 20 days fermentation. ETEC infection started at day 13. Relative abundances were determined by amplicon sequencing. Species were annotated using the RDP SeqMatch and NCBI BLAST. For species that could not unambiguously be identified at species level, the OTU identifier is displayed.

**Supplementary Figure S6. Species level microbial community composition of the mucosal gut environment in the M-SHIME.** Ileum (a) and ascending colon (b) environments under control and probiotic conditions from six different donors over the course of 20 days fermentation. ETEC infection started at day 13. Relative abundances were determined by amplicon sequencing. Species were annotated using the RDP SeqMatch and NCBI BLAST. For species that could not unambiguously be identified at species level, the OTU identifier is displayed.

**Supplementary Figure S7. Significant differences in genera level abundance between control and probiotic condition, as assessed by differential abundance analysis.** The abundance is displayed for the different gut regions from the M-SHIME. Coloured labels indicate the phylum classification of the respective genera.

**Supplementary Figure S8. Volcano plots illustrating the species (OTU numbers) significantly enriched by the probiotic treatment compared to the control in each gut niche over fermentations in the M-SHIME.** (a) ileum lumen, (b) ileum mucus, (c) ascending colon lumen, (d) ascending colon mucus. Analysis was performed from day 7 (stabilized microbiota) to 20. A positive log2 fold-change indicates a stimulation of the species in the control condition (in purple) compared to a negative log2 fold -change which indicates a stimulation of the species in the probiotic condition (in blue). The log transformed adjusted p-value is displayed on the y-axis and the α = 0.05 significance level is indicated by a dashed line. Only the species with an absolute log2 fold-change value exceeding 1.2 are represented, as determined by differential abundance analysis.

**Supplementary Figure S9.** **Significant differences in genera level abundance between pre- and post-infection periods under control and probiotic conditions, as assessed by differential abundance analysis.** The abundance is displayed for the different gut regions from the M-SHIME. Coloured labels indicate the phylum classification of the respective genera.

**Supplementary Figure S10.** **Volcano plots illustrating the species (OTU numbers) significantly enriched between the pre- and post-ETEC infection periods according to the gut niches in the control (a-d) and probiotic (e-f) conditions.** (a/e) ileum lumen, (b/f) ileum mucus, (c/g) ascending colon lumen, (d/h) ascending colon mucus. The pre-infection period is defined on days 7-12 and the post-infection period on days 13-20. A positive log_2_ fold-change indicates a stimulation of the species in the pre-infection (in grey) compared to a negative log_2_ fold-change which indicates a stimulation of the species in the post-infection (in brown). The log transformed adjusted p-value is displayed on the y-axis and the α = 0.05 significance level is indicated by a dashed line. Only the species with an absolute log_2_ fold-change value exceeding 1.2 are represented, as determined by differential abundance analysis.
